# Body mass index does not decline during winter for the sedentary marine gastropod *Crepidula fornicata*

**DOI:** 10.1098/rsbl.2023.0026

**Published:** 2023-06-14

**Authors:** Amanda M. Franklin, Alberto Rivera, Justin Robbins, Jan A. Pechenik

**Affiliations:** ^1^ School of BioSciences, The University of Melbourne, Parkville, Victoria 3010, Australia; ^2^ Biology Department, Tufts University, Medford, MA 02155, USA; ^3^ Department of Environmental and Ocean Sciences, University of San Diego, San Diego, CA 92110, USA; ^4^ Forestry and Environmental Conservation Department, Clemson University, Clemson, SC 29634, USA

**Keywords:** winter biomass changes, chlorophyll, temperature, gastropods, slippershell snails

## Abstract

Seasonal extremes in environmental conditions can substantially limit the growth and reproduction of animals. Sedentary marine animals are particularly susceptible to winter food limitation since they cannot relocate to more favourable conditions. In several temperate-zone bivalve species, substantial winter tissue mass declines have been documented; however, no comparable studies have been conducted on intertidal gastropods. Here, we investigate whether the suspension-feeding intertidal gastropod *Crepidula fornicata* also loses substantial tissue mass during the winter. We calculated body mass index (BMI) for individuals collected in New England at different times of year for 7 years to determine whether BMI declines through winter or varies seasonally. Remarkably, *C. fornicata* body mass did not decline significantly during winter months; indeed, a relatively poorer body condition was associated with higher seawater temperature, higher air temperature and higher chlorophyll concentration. In a laboratory experiment, we found that *C. fornicata* adults that were not fed for three weeks at 6°C (local winter seawater temperature) showed no detectable declines in BMI compared to field-collected individuals. Future studies should document energy budgets of *C. fornicata* and other sedentary marine animals at low winter seawater temperatures, and the impact of short-term elevated temperatures on those energy budgets.

## 1. Introduction

Seasonal variation in environmental conditions, such as temperature or food availability, can negatively impact growth, fecundity and in severe cases, the survival of animals [1–5]. To mitigate the effects of seasonal changes, animals use a suite of behavioural, physiological and morphological adaptations [6–10]. These adaptations vary among individuals, populations and species, and thus so do the impact of seasonal changes [10–13]. By documenting how various organisms respond to seasonal changes, we can identify different adaptations to challenging environmental conditions and improve predictions of how changing climates will impact ecosystems [14–16].

In temperate marine habitats, winter can bring challenging conditions, such as low seawater temperatures and low food availability. Sessile suspension-feeders are particularly susceptible to these conditions, in part because they are unable to relocate to more favourable microhabitats. Numerous studies have documented substantial declines in tissue weight for various temperate marine bivalves during the winter, including both burrowing species and those attached to rocks [4,17–25]. For example, from early autumn to spring, the dry tissue weight of four bivalve species sampled in the Dutch Wadden Sea declined by up to 50% [18,21]. While documented declines are often greater in warmer winters [4,19,21], likely related to higher rates of energy expenditure in the absence of a sufficient food supply [25], they were still present and substantial in average or cooler winters.

Intertidal suspension-feeding organisms may be especially impacted by winter conditions because they are emersed during low tide twice per day and unable to feed while exposed to air. However, studies investigating winter tissue mass declines have only been conducted for bivalves and do not seem to have been conducted with other intertidal animals. In the present study, we sought to determine whether the marine gastropod *Crepidula fornicata* also shows pronounced losses of tissue weight during New England winters, and, if so, whether it might reflect nutritional inadequacy. As with most bivalve species, *C. fornicata* juveniles and adults are suspension-feeders and live a largely sedentary life [26–28], although some individuals may be attached to loose rocks that can be tossed around with water movement. Here, we use 7 years of intertidal field collections to assess seasonal changes in adult body mass index (BMI) and whether BMI declines through the winter. We also conducted a three-week laboratory study to examine whether a lack of food at winter temperatures results in weight loss.

## 2. Material and methods

### (a) Field study: seasonal changes in body mass index

*Crepidula fornicata* are found intertidally and subtidally [27,29,30] in semi-permanent stacks [26–28,31] along the East Coast of the USA from Florida to Canada [32]. We collected stacks of *C. fornicata* from the low to mid-intertidal zone in different seasons at low tide from Nahant, Massachusetts; Beverly, Massachusetts; and Wickford, Rhode Island, USA, in 2009, 2010, 2014 and each succeeding year from 2016 to 2019 (7 years in total, over a 10-year period). Animals were transported to the laboratory in a cooler and shell length was recorded to the nearest 0.1 mm. Dry tissue weight was determined following methods described by Pechenik *et al*. [33].

Following the approach in previous research [4], for each individual (*n* = 670), we quantified body condition using a BMI:

$$\mathrm{BMI}=\frac{\mathrm{mg}\;\mathrm{dry}\;\mathrm{tissue}\;\mathrm{weight}}{{\left(\mathrm{cm}\;\mathrm{shell}\;\mathrm{length}\right)}^{3}}\;.$$

It is difficult to identify male and female *C. fornicata* based on morphological characteristics except during the breeding season. However, *C. fornicata* males are generally smaller than females, and there is limited size overlap between members of the two sexes [31]. Therefore, we used shell length to estimate sex, with individuals less than 25 mm in shell length classified as males (*n* = 380) and individuals greater than 30 mm in shell length classified as females (*n* = 194) [31]. To avoid any sexual misclassifications, those with shell lengths between 25 and 30 mm were classified as ‘ambiguous’ (*n* = 96; electronic supplementary material, figure S1).

To determine whether *C. fornicata* BMI changes with environmental conditions, we compiled monthly averages of sea surface temperature, air temperature and chlorophyll concentration for our sampling months [34–36]. These parameters were chosen because temperature will typically influence metabolic rate while chlorophyll A can be used as an estimator of primary productivity and therefore food availability [37]. Due to the moderate to high correlation between these parameters, we conducted a principal components analysis on the scaled variables and selected the principal component/s that had eigenvalues greater than 1 (here, PC1) for the subsequent analysis.

### (b) Laboratory study: effect of no food on body mass index

To assess whether *C. fornicata* lose weight at winter seawater temperatures in the absence of food, we conducted three replicate laboratory experiments. For each experiment, snails were collected at low tide from either Lynch Park in Beverly, MA (February 2016) or Nahant Massachusetts (March 2018 and March 2022), transported to the laboratory in a cooler, and immediately placed into an aquarium containing aerated, artificial seawater (Instant Ocean, 30 ppt) in a walk-in cold-room at 6°C, similar to April seawater temperatures in New England [34]. One third of the snails were removed from their shells within 24 h of their collection and their dry tissue weights were determined [33]. The remaining snails were placed into individual cups containing 50 ml of aerated Instant Ocean (30 ppt) at 6°C and allocated to one of two feeding treatments: (i) no food for three weeks or (ii) fed daily with Reeds Shellfish Diet 1800 *ad libitum*. Seawater was changed with freshly aerated artificial seawater (Instant Ocean) every 1–2 days. After three weeks, shell length and dry tissue weight were determined for all individuals. See electronic supplementary material, table S1 for snail size variation and sample sizes. No individuals died during the three-week period.

### (c) Statistical analyses

All analyses were conducted in R v4.1.2 [38]. To assess whether BMI correlates with environmental variables, we ran a linear model with BMI as the response variable and PC1, sex, cumulative month and the interaction between PC1 and sex as fixed effects. Cumulative month was included to account for any overall increase or decrease in BMI throughout the sampling period. We also investigated whether the BMI for *C. fornicata* changed during the winter months, using a linear model with month (October through to April) and winter year (2009/2010, 2016/2017, 2017/2018) as fixed effects. For this analysis, we only used data from years in which we had multiple collection dates throughout winter, which limited our data to that for individuals collected at Nahant.

For the laboratory experiment, to determine whether BMI differed among the treatment groups (field collected, fed in the laboratory or not fed in the laboratory), we ran a linear model. BMI was the response variable and treatment, experiment year (2016, 2018 or 2022) and the interaction between these factors were the predictor variables. Diagnostic plots indicated that none of the models violated any assumptions. To assess the significance of each factor, we compared reduced models to full models using likelihood ratio tests.

## 3. Results

Environment PC1 explained 82% of the variation in the environmental variables examined (sea surface temperature, air temperature and chlorophyll concentration). PC1 loaded strongly and positively with all variables. Therefore, positive values of PC1 indicate warm air temperatures, warm water temperatures and higher levels of chlorophyll, characteristic of summer and autumn conditions (electronic supplementary material, figure S2).

**Figure 1.**
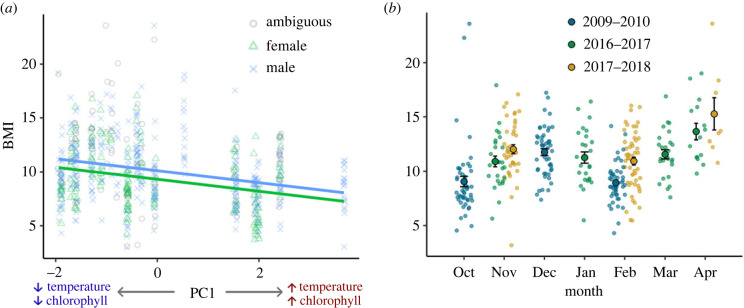
Field study: seasonal changes in BMI for *Crepidula fornicata* collected over a 10-year period. (*a*) BMI tended to decrease regardless of sex (males: blue; females: green; ambiguous: grey) with increasing PC1. Higher values of PC1 indicate warmer sea surface temperature, warmer air temperature and higher seawater chlorophyll concentration. Trend lines are from the linear model. (*b*) By month, BMI remained consistent from mid-autumn through winter until early spring. BMI was calculated as: milligrams of dry tissue weight per (centimetre of shell length)^3^.

The BMI of *C. fornicata* decreased significantly as PC1 increased (*χ*² = 43.89, d.f. = 1, *p* < 0.001), indicating that the snails were in relatively poorer condition when water and air temperatures were warmer and chlorophyll concentrations were higher (figure 1*a*). This trend was similar for both males and females (interaction term: *χ*² = 0.34, d.f. = 2, *p* = 0.84), but, on average, males were in slightly better condition than females (*χ*² = 30.30, d.f. = 2, *p* < 0.001; figure 1*a*). We detected no significant effect of cumulative month on BMI, suggesting that average BMI did not increase or decrease over the 10-year sampling period (*χ*² = 0.58, d.f. = 1, *p* = 0.45).

We found no significant change in *C. fornicata* BMI throughout the winter (*χ*² = 2.24, d.f. = 1, *p* = 0.13; figure 1*b*). This result was consistent for both males and females (interaction term: *χ*² = 3.55, d.f. = 1, *p* = 0.17), but, on average, males were in slightly better condition than females (*χ*² = 7.12, d.f. = 1, *p* = 0.03). *Crepidula fornicata* collected in the winters of 2016–2017 and 2017–2018 had similar BMI, and their mean BMI was 18% higher on average than it was for the *C. fornicata* individuals that had been collected in the winter of 2009–2010 (*χ*² = 23.82, d.f. = 2, *p* < 0.001; figure 1*b*).

**Figure 2.**
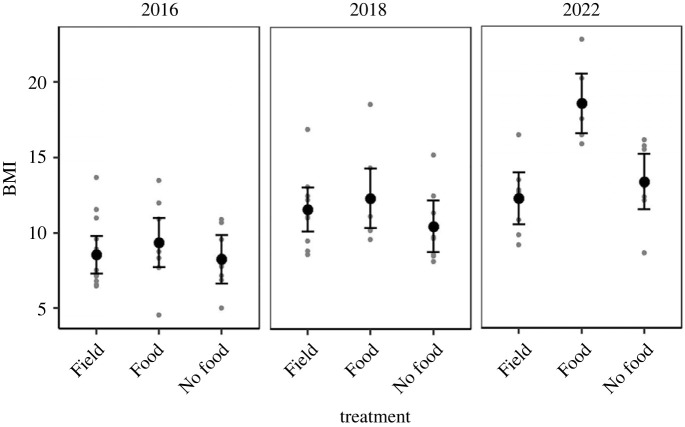
Laboratory study: the impact of three weeks without food at 6°C on the BMI of *Crepidula fornicata* adults. In 2022, the snails used in this study were collected over a narrow size range, to reduce effects of size on the results. ‘Field’ indicates snails that were measured and prepared for dry tissue weight shortly after being collected from the field, ‘Food’ indicates those that were fed daily in the laboratory and ‘No food’ indicates those that were not fed throughout the three-week experiment.

In all laboratory experiments, individuals that were fed during the three-week maintenance period at 6°C marginally increased their BMI compared to field-collected snails and snails that were not fed for three weeks (*χ*² = 12.82, d.f. = 2, *p* = 0.002; figure 2). This effect was largely driven by the 2022 experiment that used snails within a narrow size range (interaction term: *χ*² = 13.91, d.f. = 4, *p* = 0.008; figure 2). Across all experiments, there was no difference in BMI between field-collected snails and snails that were not fed for three weeks, suggesting that a lack of food at winter temperatures does not lead to a substantial decline in biomass for this species, for at least three weeks.

## 4. Discussion

For many sedentary marine organisms, winter conditions lead to substantial declines in tissue weight as high as 70% (for examples see: [4,17–19,22]). However, here we have shown that the marine gastropod *C. fornicata* showed no detectable decline in body condition throughout the winter at our study sites. Instead, BMI was lower during the summer months, when temperatures are warmer and chlorophyll concentrations are higher. Furthermore, we detected no decline in BMI for *C. fornicata* adults maintained in the laboratory at winter temperatures even after three weeks without food, suggesting low-energy requirements in winter. These results suggest that *C. fornicata* from our studied New England populations do not experience the substantial negative impacts from winter conditions that many bivalve species do. This emphasizes the importance of studying different organisms to identify different degrees of adaptation to challenging environmental conditions.

Our results are unexpected, considering that New England winters are characterized by low primary productivity, and that intertidal individuals are unable to feed for several hours per day during emersion at low tide. Together, the field and laboratory studies suggest that rates of energy expenditure must be extremely low for this species under winter conditions. This idea is supported by previous research that found respiration rates of *C. fornicata* are about 90% lower at 5°C than at 10°C [39]. At our study sites, winter seawater temperatures may be as low as 3.5°C [33], suggesting energy expenditure in the field may be even lower than in our laboratory studies. *Crepidula fornicata* are located over a large latitudinal range, from southern USA to southern Norway [40], and similar studies could be conducted to investigate intraspecific variation in adaptation to winter conditions.

We found that BMI for intertidal *C. fornicata* decreased slightly as temperatures and chlorophyll concentrations increased in summer and early autumn compared with the BMI of individuals collected in the winter. During summer and autumn, water and air temperatures are higher than in winter and spring, and higher temperatures can increase respiration rates of intertidal organisms [41]. Therefore, metabolic demands may outstrip food supply in summer and autumn and/or food supply may outstrip metabolic demands in winter and spring. In addition, *C. fornicata* gonadal development begins during early spring and reproduction occurs during mid-spring until mid-autumn [42]. It is possible that *C. fornicata* may prioritize investment into reproduction rather than somatic tissue in mid-summer to mid-autumn, resulting in a lower BMI. These underlying mechanisms require further investigation.

Substantial winter weight loss has been documented in several bivalve species; these losses were especially large when winter water temperatures were even just a few degrees higher than normal [4,21,25,43]. This likely reflects higher rates of energy expenditure in the absence of a sufficient food supply [4,44,45]. Periods of unusually high winter air temperatures seem to have become more common in New England in recent years [33], which could eventually lead to winter weight loss for intertidal *C. fornicata* as well. To determine the effects of short-term elevated thermal stress, future studies should document winter energy budgets of *C. fornicata* and other intertidal animals, and the impact of periodic short-term elevated temperatures on metabolic rates, digestive functioning and assimilation efficiencies. Such research will help to determine how intertidal communities will respond to climate change in the future.

## Data Availability

Data and analysis code are available from the Dryad Digital Repository: https://doi.org/10.5061/dryad.msbcc2g2r [46]. Additional figures and tables are provided in the electronic supplementary material [47].

## References

[1] Rimbach R, Pillay N, Schradin C. 2021 Prolonged growth during the food-restricted dry season in a small African mammal. J. Mammal. **102**, 296-307. (10.1093/jmammal/gyaa169)

[2] Zylstra ER, Ries L, Neupane N, Saunders SP, Ramírez MI, Rendón-Salinas E, Oberhauser KS, Farr MT, Zipkin EF. 2021 Changes in climate drive recent monarch butterfly dynamics. Nat. Ecol. Evol. **5**, 1441-1452. (10.1038/s41559-021-01504-1) 34282317

[3] Abarca M, Larsen EA, Ries L. 2019 Heatwaves and novel host consumption increase overwinter mortality of an imperiled wetland butterfly. Front. Ecol. Evol. **7**, 193. (10.3389/fevo.2019.00193)

[4] Honkoop P, Beukema J. 1997 Loss of body mass in winter in three intertidal bivalve species: an experimental and observational study of the interacting effects between water temperature, feeding time and feeding behaviour. J. Exp. Mar. Biol. Ecol. **212**, 277-297. (10.1016/S0022-0981(96)02757-8)

[5] Benard MF. 2015 Warmer winters reduce frog fecundity and shift breeding phenology, which consequently alters larval development and metamorphic timing. Glob. Change Biol. **21**, 1058-1065. (10.1111/gcb.12720) 25263760

[6] Kanciruk P, Herrnkind W. 1978 Mass migration of spiny lobster, *Panulirus argus* (Crustacea: Palinuridae): behavior and environmental correlates. Bull. Mar. Sci. **28**, 601-623.

[7] Rebach S. 1974 Burying behavior in relation to substrate and temperature in the hermit crab, *Pagurus longicarpus*. Ecology **55**, 195-198. (10.2307/1934636)

[8] Kingsolver JG, Moffat RJ. 1982 Thermoregulation and the determinants of heat transfer in *Colias* butterflies. Oecologia **53**, 27-33. (10.1007/BF00377132) 28310599

[9] Tattersall GJ, Sinclair BJ, Withers PC, Fields PA, Seebacher F, Cooper CE, Maloney SK. 2012 Coping with thermal challenges: physiological adaptations to environmental temperatures. Comprehen. Physiol. **2**, 2151-2202. (10.1002/cphy.c110055) 23723035

[10] Franklin AM, Rankin KJ, Hugall A, Stuart-Fox D. 2022 Exposure to thermal extremes favors higher solar reflectivity in intertidal gastropods. Iscience **25**, 105674. (10.1016/j.isci.2022.105674) 36536676 PMC9758529

[11] Wang LY, Franklin AM, Black JR, Stuart-Fox D. 2021 Heating rates are more strongly influenced by near-infrared than visible reflectance in beetles. J. Exp. Biol. **224**, jeb242898. (10.1242/jeb.242898) 34494652

[12] Forsman A. 2000 Some like it hot: intra-population variation in behavioral thermoregulation in color-polymorphic pygmy grasshoppers. Evol. Ecol. **14**, 25-38. (10.1023/A:1011024320725)

[13] Muñoz MM, Bodensteiner BL. 2019 Janzen's hypothesis meets the Bogert Effect: connecting climate variation, thermoregulatory behavior, and rates of physiological evolution. Integr. Organ. Biol. **1**, oby002. (10.1093/iob/oby002) PMC7671085 33791511

[14] Kearney M, Shine R, Porter WP. 2009 The potential for behavioral thermoregulation to buffer 'cold-blooded' animals against climate warming. Proc. Natl Acad. Sci. USA **106**, 3835-3840. (10.1073/pnas.0808913106) 19234117 PMC2656166

[15] Radchuk V et al. 2019 Adaptive responses of animals to climate change are most likely insufficient. Nat. Commun. **10**, 3109. (10.1038/s41467-019-10924-4) 31337752 PMC6650445

[16] Seebacher F, White CR, Franklin CE. 2015 Physiological plasticity increases resilience of ectothermic animals to climate change. Nat. Clim. Change **5**, 61-66. (10.1038/nclimate2457)

[17] Ansell A. 1974 Seasonal changes in biochemical composition of the bivalve *Chlamys septemradiata* from the Clyde Sea area. Mar. Biol. **25**, 85-99. (10.1007/BF00389258)

[18] Beukema J, De Bruin W. 1977 Seasonal changes in dry weight and chemical composition of the soft parts of the tellinid bivalve *Macoma balthica* in the Dutch Wadden Sea. Neth. J. Sea Res. **11**, 42-55. (10.1016/0077-7579(77)90020-5)

[19] Bayne B, Worrall C. 1980 Growth and production of mussels *Mytilus edulis* from two populations. Mar. Ecol. Prog. Ser. **3**, 317-328. (10.3354/meps003317)

[20] Zandee D, Kluytmans J, Zurburg W, Pieters H. 1980 Seasonal variations in biochemical composition of *Mytilus edulis* with reference to energy metabolism and gametogenesis. Neth. J. Sea Res. **14**, 1-29. (10.1016/0077-7579(80)90011-3)

[21] Zwarts L. 1991 Seasonal variation in body weight of the bivalves *Macoma balthica*, *Scrobicularia plana*, *Mya arenaria* and *Cerastoderman edule* in the Dutch Wadden Sea. Neth. J. Sea Res. **28**, 231-245. (10.1016/0077-7579(91)90021-R)

[22] Beninger PG, Lucas A. 1984 Seasonal variations in condition, reproductive activity, and gross biochemical composition of two species of adult clam reared in a common habitat: *Tapes decussatus* L. (Jeffreys) and *Tapes philippinarum* (Adams & Reeve). J. Exp. Mar. Biol. Ecol. **79**, 19-37. (10.1016/0022-0981(84)90028-5)

[23] Ahn IY, Surh J, Park YG, Kwon H, Choi KS, Kang SH, Choi HJ, Kim KW, Chung H. 2003 Growth and seasonal energetics of the Antarctic bivalve *Laternula elliptica* from King George Island, Antarctica. Mar. Ecol. Prog. Ser. **257**, 99-110. (10.3354/meps257099)

[24] Fitzgerald-Dehoog L, Browning J, Allen BJ. 2012 Food and heat stress in the California mussel: evidence for an energetic trade-off between survival and growth. Biol. Bull. **223**, 205-216. (10.1086/BBLv223n2p205) 23111132

[25] Waldeck P, Larsson K. 2013 Effects of winter water temperature on mass loss in Baltic blue mussels: implications for foraging sea ducks. J. Exp. Mar. Biol. Ecol. **444**, 24-30. (10.1016/j.jembe.2013.03.007)

[26] Conklin EG. 1898 Environmental and sexual dimorphism in *Crepidula*. Proc. Acad. Nat. Sci. Phila **50**, 435-444.

[27] Diederich CM, Pechenik JA. 2013 Thermal tolerance of *Crepidula fornicata* (Gastropoda) life history stages from intertidal and subtidal subpopulations. Mar. Ecol. Prog. Ser. **486**, 173-187. (10.3354/meps10355)

[28] Dupont L, Richard J, Paulet YM, Thouzeau G, Viard F. 2006 Gregariousness and protandry promote reproductive insurance in the invasive gastropod *Crepidula fornicata*: evidence from assignment of larval paternity. Mol. Ecol. **15**, 3009-3021. (10.1111/j.1365-294X.2006.02988.x) 16911217

[29] Collin R. 2003 Evolution of mode of development in *Crepidula* (Gastropoda: Calyptraeidae): causes and consequences. PhD thesis, University of Chicago, Chicago, IL.

[30] Diederich CM, Bashevkin SM, Chaparro OR, Pechenik JA. 2015 Desiccation tolerance and lifting behavior in *Crepidula fornicata* (Gastropoda). Mar. Ecol. Prog. Ser. **528**, 235-243. (10.3354/meps11284)

[31] Coe WR. 1936 Sexual phases in *Crepidula*. J. Exp. Zool. **72**, 455-477. (10.1002/jez.1400720306)

[32] Collin R. 2001 The effects of mode of development on phylogeography and population structure of North Atlantic *Crepidula* (Gastropoda: Calyptraeidae). Mol. Ecol. **10**, 2249-2262. (10.1046/j.1365-294X.2001.01372.x) 11555267

[33] Pechenik JA, Chaparro OR, Lazarus ZM, Tellado GV, Ostapovich EM, Clark D. 2020 Impact of short-term elevated temperature stress on winter-acclimated individuals of the marine gastropod *Crepidula fornicata*. Mar. Environ. Res. **162**, 105180. (10.1016/j.marenvres.2020.105180) 33126112

[34] NASA Earth Observations. 2022 Sea surface temperature (1 month – AQUA/MODIS). See https://neo.gsfc.nasa.gov/ (accessed June 2022).

[35] NASA Earth Observations. 2022 Chlorophyll concentration (1 month – AQUA/MODIS). See https://neo.gsfc.nasa.gov/ (accessed June 2022).

[36] NOAA National Centers for Environmental information. 2022 Climate at a glance: city time series. See https://www.ncdc.noaa.gov/cag/ (accessed June 2022).

[37] Huot Y, Babin M, Bruyant F, Grob C, Twardowski M, Claustre H. 2007 Does chlorophyll a provide the best index of phytoplankton biomass for primary productivity studies? Biogeosci. Disc. **4**, 707-745.

[38] R Core Team. 2021 R: a language and environment for statistical computing. Vienna, Austria: R Foundation for Statistical Computing.

[39] Newell R, Kofoed LDB. 1977 The energetics of suspension-feeding in the gastropod *Crepidula fornicata* L. J. Mar. Biol. Assoc. U. K. **57**, 161-180. (10.1017/S0025315400021317)

[40] Pechenik JA, Diederich CM, Browman HI, Jelmert A. 2017 Fecundity of the invasive marine gastropod *Crepidula fornicata* near the current northern extreme of its range. Invertebr. Biol. **136**, 394-402. (10.1111/ivb.12194)

[41] Newell RC. 1969 Effect of fluctuations in temperature on the metabolism of intertidal invertebrates. Am. Zool. **9**, 293-307. (10.1093/icb/9.2.293)

[42] Richard J, Huet M, Thouzeau G, Paulet YM. 2006 Reproduction of the invasive slipper limpet, *Crepidula fornicata*, in the Bay of Brest, France. Mar. Biol. **149**, 789-801. (10.1007/s00227-005-0157-4)

[43] Beukema J, Dekker R, Jansen J. 2009 Some like it cold: populations of the tellinid bivalve *Macoma balthica* (L.) suffer in various ways from a warming climate. Mar. Ecol. Prog. Ser. **384**, 135-145. (10.3354/meps07952)

[44] Beukema J, Dekker R. 2020 Half a century of monitoring macrobenthic animals on tidal flats in the Dutch Wadden Sea. Mar. Ecol. Prog. Ser. **656**, 1-18. (10.3354/meps13555)

[45] Melzner F, Buchholz B, Wolf F, Panknin U, Wall M. 2020 Ocean winter warming induced starvation of predator and prey. Proc. R. Soc. B **287**, 20200970. (10.1098/rspb.2020.0970) PMC7423679 32673558

[46] Franklin AM, Rivera A, Robbins J, Pechenik JA. 2023 Data from: Body mass index does not decline during winter for the sedentary marine gastropod *Crepidula fornicata*. Dryad Digital Repository. (10.5061/dryad.msbcc2g2r) PMC10264102 37311546

[47] Franklin AM, Rivera A, Robbins J, Pechenik JA. 2023 Body mass index does not decline during winter for the sedentary marine gastropod *Crepidula fornicata*. Figshare. (10.6084/m9.figshare.c.6662932) PMC10264102 37311546

